# What is the rate and degree of deformity in femoral diaphyseal nonunion? A CT-based retrospective cohort study

**DOI:** 10.1007/s00068-026-03314-1

**Published:** 2026-08-28

**Authors:** Joep Nabben, Robert K. Wagner, Floor Groepenhoff, Tim A. Verwest, Yoram Bont, Peter Kloen, Stein J. Janssen

**Affiliations:** 1 Department of Orthopaedic Surgery and Sports Medicine, Amsterdam UMC, location Meibergdreef 9, Amsterdam, the Netherlands; 2 Amsterdam Movement Sciences, Musculoskeletal Health, Amsterdam, the Netherlands; 3 Department of Orthopaedic Surgery, Massachusetts General Hospital, Boston, MA USA; 4 Harvard Medical School Orthopaedic Trauma Initiative, Boston, MA USA; 5 Department of Radiology and Nuclear Medicine, Amsterdam UMC, location meibergdreef 9, Amsterdam, the Netherlands

**Keywords:** Femoral shaft nonunion, Malalignment, Torsional deformity, Computed tomography, Femoral shaft fractures, Leg length discrepancy

## Abstract

**Background:**

This study investigated the rate and degree of deformity in femoral diaphyseal nonunion using preoperative bilateral CT-scans.

**Methods:**

A retrospective cohort study was conducted at a single Level 1 trauma center. Patients aged > 18 years who underwent operative treatment for femoral diaphyseal nonunion between 2015 and 2025 and had preoperative bilateral femur CT-scans were included. Axial, sagittal, coronal, and length differences between the femoral nonunion and the contralateral healthy femur were measured. Torsional deformity ≥ 15° and length discrepancy ≥ 20 mm were considered clinically relevant. Thresholds of ≥ 5° and ≥ 10° were used to identify outliers in coronal and sagittal plane deformity, respectively.

**Results:**

Thirty-two patients met the inclusion criteria (median age 57 years, 63% male). Torsional deformity ≥ 15° was present in 10 patients (31%), including internal rotation in 4 (13%) and external rotation in 6 (19%). Sagittal plane deformity ≥ 10° was observed in 5 patients (16%), with apex anterior angulation in 1 (3%) and apex posterior angulation in 4 (13%). Mean sagittal plane angulation was 9.7° ± 6.7° in the affected limb and 12.5° ± 2.6° in the unaffected limb (*p* = 0.017). Coronal plane deformity ≥ 5° was present in 10 patients (31%), including varus in 8 (25%) and valgus in 2 (6%). Mean coronal angulation was 5.3° ± 6.0° in the affected limb and 3.3° ± 2.5° in the unaffected limb (*p* = 0.099). Mean femoral length was 439 ± 31 mm in the affected limb and 445 ± 28 mm in the unaffected limb (*p* = 0.015), with a length discrepancy ≥ 20 mm in 5 patients (16%).

**Conclusions:**

Clinically relevant torsional deformity was present in one in three patients undergoing femoral shaft nonunion surgery, while femoral length discrepancy ≥ 20 mm occurred in one in six. A small but significant decrease in apex anterior angulation was observed, and most coronal plane outliers were in varus. These findings underline the importance of recognizing deformity in femoral nonunion.

**Supplementary Information:**

The online version contains supplementary material available at https://doi.org/10.1007/s00068-026-03314-1.

## Introduction

Femoral shaft nonunions are severe complications with important impact on general and physical health [1]. Several factors are known to contribute to the development of nonunion, including host factors (e.g., diabetes mellitus and smoking), and local factors (e.g., infection, unstable osteosynthesis) [2]. The treatment for femoral shaft nonunion is based on revision surgery, and a variety of treatment options exist. The basic principles for treating most femoral shaft nonunion consist of finding and treating the infection (if present), removal of interposed fibrous tissue and/or dead bone, removal of hardware, recreating continuity of the medullary canal, correction of any possible deformity, and subsequently creating a stable osteosynthesis under compression. Bone graft (autologous/homologous or a substitute) is added for most oligotrophic and atrophic nonunions, whereas in hypertrophic nonunions that may not be necessary. Fixation after previous nailing is often done by reamed exchange nailing with or without plate augmentation, but is dependent on nonunion location and preference of the surgeon [1, 3].

The correction of deformity is an important aspect of nonunion treatment, as residual or a new deformity may contribute to an unstable mechanical environment resulting in a nonunion [4–8]. In addition, deformities may be related to long-term sequelae such as development of osteoarthritis, as well as stress fractures [4]. While clinical assessment and radiographs can give an indication of the degree and direction of deformity; the gold standard for identifying deformity in all three planes is a bilateral CT-scan of both femurs [9]. Nevertheless, obtaining bilateral complete femoral CT-scans in femoral nonunion is not universally part of clinical practice, which may lead to underdiagnosis and subsequent undertreatment of deformities at time of nonunion correction. Obtaining a bilateral CT-scan may be especially important for assessment of axial (torsion) deformity, as this is more difficult to detect on plain anteroposterior and lateral radiographs.

The primary aim of this study was to assess the rate and extent of torsional deformity in femoral diaphyseal nonunion. The secondary aim was to determine sagittal (apex anterior/ apex posterior angulation) and coronal plane (varus/valgus) deformities and femoral length differences in femoral diaphyseal nonunion.

## Methods

### Patients

All adult patients who underwent operative treatment for a femoral shaft nonunion and who had a preoperative bilateral CT-scan of the complete femur available between 2015 and 2025 were retrospectively identified from an urban Level 1 Trauma and tertiary care referral center for orthopaedic trauma institutional database. Exclusion criteria were: (1) ipsilateral or contralateral hip or knee prostheses (as this obscures measurements), (2) previous fractures of the contralateral limb, (3) nonunions from pathological fractures, (4) patients with nonunions from osteotomies and (5) femoral neck, head and distal intra-articular femoral nonunions. This study was approved by the local Medical Ethical Review Committee (2024.0225) and all patients signed prior to start of the study informed consent to use their data for research. The study was conducted in line with the STROBE (Strengthening the Reporting of Observational Studies in Epidemiology) protocol.

### Data collection

Data for this study was retrospectively extracted from electronic medical records. Demographic data included age, gender, body mass index (BMI), American Society of Anesthesiologists classification (ASA), smoking status, and diabetes status [10]. Nonunion characteristics included the time from the initial injury to the nonunion surgery in months; injury mechanism classified as either low or high-energy accidents, the initial AO/OTA subclassification (type A [simple fractures], type B [wedge fractures], and type C [comminuted fractures]), and whether there were signs of infection at the fracture site [11–13]. The type of fixation construct present at time of nonunion repair was categorized into intramedullary nail (IMN), single plate, nail-plate combinations, or dual plate combinations. The presence of construct failure was recorded and categorized as: broken or loose screws, broken plate(s), or broken nail.

### CT-measurements

Measurements were independently performed by three observers (including two residents specializing in musculoskeletal radiology and a research fellow) using Sectra IDS7 radiological software. Torsional deformity was determined by the angle between a line drawn across the femoral neck and the posterior aspect of distal femoral condyles of both femurs (Online Resource 1) [14]. Clinically relevant torsional deformities of the femur are often defined as a difference in version of 15 degrees or more compared with the contralateral side due to the clinical complaints that are related to this degree of malrotation [14–16]. Therefore, the following malrotation categories were used: < 9 degrees (no/minimal torsional deformity); 10–14 degrees (borderline torsional deformity); ≥ 15 degrees (torsional deformity).

The degree of sagittal (apex anterior / apex posterior angulation) and coronal plane deformity (varus/valgus) was assessed by the angle between the central lines of the proximal fracture segment and the distal fracture segment (Online Resource 2 and 3). Proximal and distal reference lines were placed along which the central lines were drawn to ensure the most accurate measurements possible. Positive values indicate apex anterior angulation or varus, and negative values indicate apex posterior angulation or valgus [17, 18]. To the best of current knowledge, no published literature defines thresholds at which coronal or sagittal plane femoral shaft deformity should be considered clinically relevant. Therefore, for the purpose of outlier identification, coronal-plane angulation was defined as a difference of ≥ 5° (varus or valgus) and sagittal-plane angulation (apex-anterior/posterior) as ≥ 10° between the affected and unaffected limb, based on the distribution of coronal and sagittal plane angulation values reported in the literature [19–21].

Femoral length difference was determined bilaterally by measuring the length between the most proximal part of the trochanter major and most distal part of femoral condyles (Online Resource 4) [22]. Clinically relevant length differences of the femur are defined as a difference in length of 20 mm or more; and therefore the following categories were used ≤ 9 mm (no/minimal leg length discrepancy); 10–19 mm (some leg length discrepancy); ≥ 20 mm (moderate to substantial leg length discrepancy) [23].

### Statistical analysis

Descriptive statistics are reported using median with interquartile range (IQR) for continuous variables and mean with standard deviation (SD) depending on data distribution, and as frequencies with percentages for categorical variables. Differences in outcome variables (deformities and femoral length) between the affected and unaffected limb were assessed using paired t-test or Wilcoxon signed rank test, depending on data distribution. In addition, to evaluate whether the type of hardware was associated with the degree of torsional deformity, patients were divided into two groups: those treated with an intramedullary nail and those treated with a plate. Comparisons between these groups were performed using an independent t-test. A one-way ANOVA was conducted to assess the relationship between AO/OTA fracture classification and the severity of torsional deformity. To assess interobserver reliability, the intraclass correlation coefficient (ICC) was calculated for all measurements using a two-way random effects model with absolute agreement. Interpretation of ICC values followed the guidelines with values < 0.5 indicating poor, 0.5–0.75 moderate, 0.75–0.9 good, and > 0.9 excellent reliability [24]. The ICC showed moderate to excellent reliability and therefore measurements were averaged for further analysis (Online Resource 5). A two-tailed p-value of less than 0.05 was considered statistically significant. Statistical analysis was performed using IBM SPSS statistics 28 software and R (Version 4.5.2).

## Results

### Baseline demographics

Of 94 patients assessed for eligibility, 62 were excluded due to: incomplete CT imaging (*n* = 26), presence of a prosthesis (*n* = 14), osteotomy (*n* = 7), bilateral fractures (*n* = 5), distal articular fractures (*n* = 2), pathological fractures (*n* = 4), or proximal neck or head fractures (*n* = 4). The remaining 32 patients were included as the final study population.

The median age was 57 (IQR: 34–70) years and 20/32 (63%) were male. The median BMI was 28 (IQR: 26–30). The median time from the initial injury to nonunion surgery was 19 (IQR: 11–22) months. Twenty-three patients (72%) suffered a high-energy injury mechanism, and 4 (13%) had an open fracture initially. Ten patients (31%) had an AO/OTA type A fracture, 12 (38%) had a type B fracture, and 10 (31%) had a type C fracture. Eight patients (25%) had suggestive signs of infection at time of presentation. For the initial fixation, 17 patients (53%) were treated with an intramedullary nail, 10 (31%) with a single plate and 5 (16%) with a dual plate. Eleven (34%) patients suffered any form of hardware failure, 6 patients (19%) had broken or loose screws, 1 (3%) had a broken nail, and 5 (16%) had a broken plate (Table 1).

**Table 1:** Patient and injury characteristics

Patient characteristics	Entire cohort (*n* = 32)
Age (median, IQR)	57 (34–70)
Body Mass Index (median, IQR)	28 (26–30)
Male, n (%)	20 (63)
ASA classification, n (%)	
I	9 (28)
II	19 (59)
III	4 (13)
Smoking, n (%)	5 (16)
former	7 (22)
Diabetes, n (%)	3 (9)
***Injury characteristics***	
High energy trauma, n (%)	23 (72)
Open fracture during initial injury, n (%)	4 (13)
Initial AO classification, n (%)	
32 A	10 (31)
32B	12 (38)
32 C	10 (31)
Signs of fracture-related infection, n (%)	8 (25)
Type of hardware present, n (%)	
IMN	17 (53)
Single plate	10 (31)
Dual plate	5 (16)
Construct failure, n (%) *	11 (34)
Broken or loose screws	6 (19)
Broken nail	1 (3)
Broken plate	5 (16)
Time from injury to nonunion-surgery (months) (median, IQR)	18.8 (10.6–22.0)

* Subcategories are not mutually exclusive; more than one type of construct failure could occur in the same patient

Missing values Body Mass Index (*n* = 1), High energy trauma (*n* = 2), Open fracture during initial injury (*n* = 1), Time from injury to nonunion-surgery (*n* = 1)

Abbreviations IQR = Inter Quartile Range ASA = American Society of Anesthesiologists, IMN = Intramedullary nail, AO = Arbeitsgemeinschaft für Osteosynthesefragen

### Femoral deformities

**Fig. 1:**
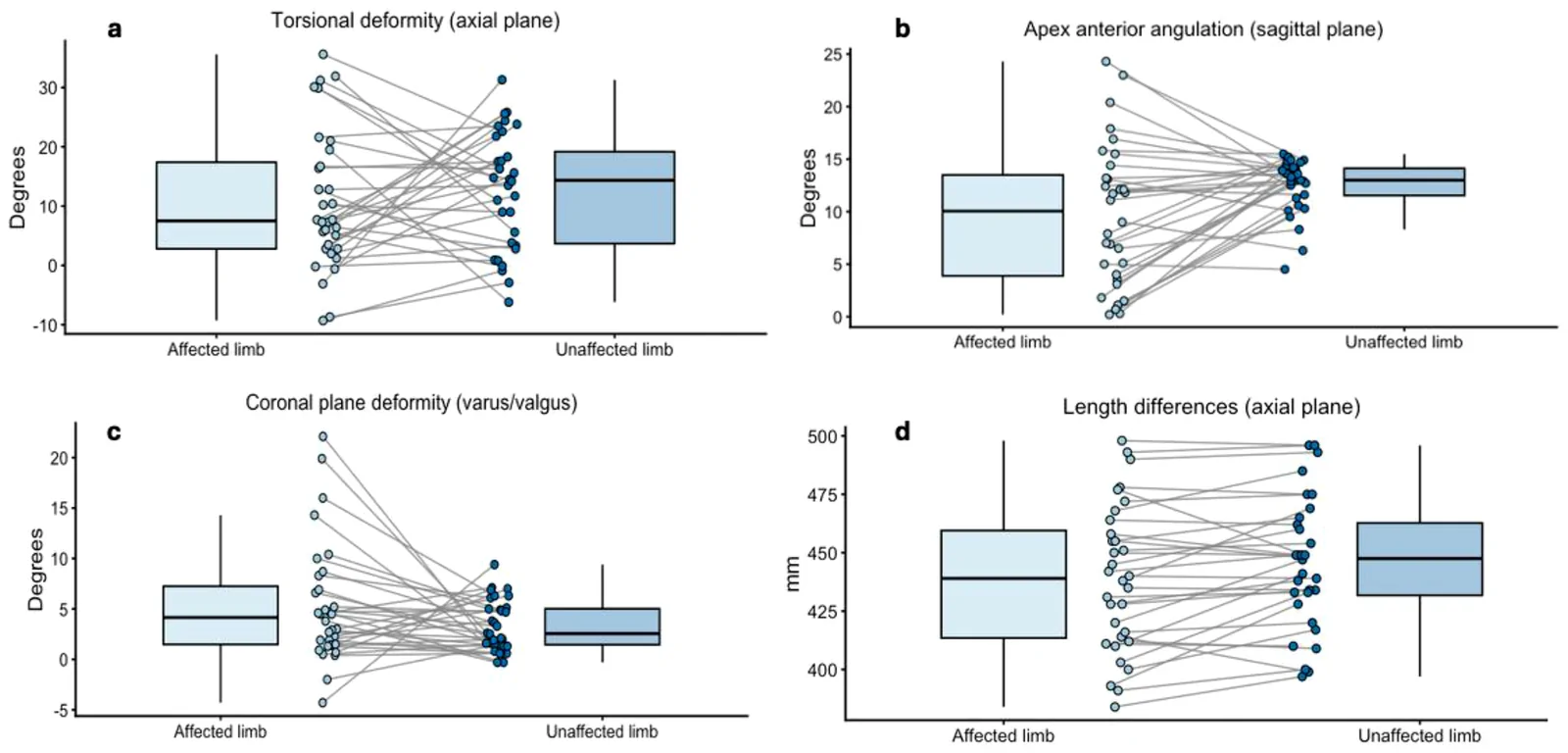
Box-and-jitter plot of (**a**) torsional deformity, (**b**) apex anterior angulation, (**c**) coronal plane deformity, and (**d**) length differences in the affected limb with the unaffected limb as reference. Positive values indicate internal rotation, anterior angulation and varus, respectively. Boxes indicate the median and interquartile range; jittered points show individual measurements, and grey lines connect paired observations within participants

Ten (31%) patients had ≥ 15° malrotation, of which 4 (13%) had ≥ 15° internal rotation, and 6 (19%) had ≥ 15° external rotation (Table 2). The mean anteversion (or internal rotation) of the affected femur was 10.7° ± 11.8°, while the mean anteversion (or internal rotation) of the unaffected femur was 12.7° ± 9.7°. This resulted in a mean degree of torsional difference of 1.9° ± 14.3° of internal/external rotation (*p* = 0.453) (Fig. 1a).

**Table 2:** Torsional deformities (axial plane) compared between affected and unaffected limb

Torsion (degrees)	Internal rotation	External rotation	*Total (n = 32)*
< 9 (no/minimal torsional deformity), n (%)	6 (19)	10 (31)	16 (50)
10–14 (borderline torsional deformity), n (%)	3 (9)	3 (9)	6 (19)
≥ 15 (torsional deformity), n (%)	4 (13)	6 (19)	10 (31)
Total, n (%)	13 (41)	19 (59)	32 (100)

Five (16%) patients had ≥ 10° difference in sagittal plane, of which 1 (3%) had apex anterior angulation and 4 (13%) had apex posterior angulation. The mean of the affected limb was 9.7° ± 6.7°, compared to 12.5° ± 2.6° in the unaffected limb (mean paired difference was − 2.8° ± 6.3°, *p* = 0.017) (Fig. 1b).

Ten (31%) patients had ≥ 5° difference in coronal plane, of which 8 (25%) had varus angulation and 2 (6%) had valgus angulation. The mean of the affected limb was 5.3° ± 6.0° varus, compared to 3.3° ± 2.5° in the unaffected limb (mean paired difference was 2.0° ± 6.7°, *p* = 0.099) (Fig. 1c).

Of the 32 patients, 5 (16%) had a femoral length discrepancy ≥ 20 mm. The mean femoral length in the affected limb was 439 ± 31 mm and 445 ± 28 mm in the unaffected limb (mean difference was − 6 ± 14 mm, *p* = 0.015) **(**Fig. 1d; Table 3**).**

**Table 3:** Femoral length differences (mm) between affected and unaffected limb

Length differences (mm)	*Total (n = 32)*
< 9 (no/minimal leg length discrepancy), n (%)	19 (59)
10–19 (some leg length discrepancy), n (%)	8 (25)
≥ 20 (moderate to substantial leg length discrepancy), n (%)	5 (16)

### Influences of type of hardware and fracture type on torsional deformities

No difference in torsional deformity was found between the groups based on the type of hardware (intramedullary nail, mean difference of 1.7° ± 14.3° vs. plate, mean difference of -6° ± 13.7°, *p* = 0.134) (Table 4).

**Table 4:** Type of hardware present in different axial deformity groups

Torsion (degrees)	*Nail (n = 17)*	*Plate(s) (n = 15)*
< 9 (no/minimal torsional deformity), n (%)	9 (53)	7 (47)
10–14 (borderline torsional deformity), n (%)	1 (6)	5 (33)
≥ 15 (torsional deformity), n (%)	7 (41)	3 (20)

Influence of type of hardware on severity of torsional deformity was assessed using an independent two-sample t-test; *p* = 0.134

### Influences of fracture type on torsional deformities

No statistically significant difference was found between the groups regarding the distribution of torsional deformities according to AO/OTA classification (*p* = 0.398) (Table 5).

**Table 5:** Distribution of rotational deformities according to AO classification

Torsion (degrees)	*Type A (n = 10)*	*Type B (n = 12)*	*Type C (n = 10)*
< 9 (no/minimal torsional deformity), n (%)	6 (60)	6 (50)	4 (40)
10–14 (borderline torsional deformity), n (%)	0 (0)	4 (33)	2 (20)
≥ 15 (torsional deformity), n (%)	4 (40)	2 (17)	4 (40)

Influence of AO/OTA classification (type A: simple fracture, type B: wedge fracture and type C comminuted fractures) on severity of torsional deformity was assessed using a one-way ANOVA; *p* = 0.398

## Discussion

Correction of deformity is an important part of nonunion treatment to improve the mechanical environment and minimize the risk of long-term sequelae. Despite its clinical relevance, deformity (pre and/or post-operative) remains an underappreciated factor in the treatment of femoral nonunion. The findings presented herein clearly demonstrate that almost one in three patients presenting with a femoral shaft nonunion had a torsional deformity of ≥ 15° compared with the contralateral limb. Furthermore, a femoral length discrepancy of ≥ 20 mm was observed in approximately 1 out of 6 patients, ≥ 10° difference in sagittal plane angulation in about 1 out of 6 patients, and ≥ 5° difference in coronal plane angulation in about 1 out of 3 patients.

The current study results suggest that torsional deformities are common in femoral diaphyseal nonunions. The findings are in line with studies describing rotational malalignment after acute femoral shaft fractures. Jaarsma et al. (2004) reported that 28% of 76 patients with isolated femoral shaft fractures treated with intramedullary nails had a torsional deformity of ≥ 15° [25]. Bråten et al. observed that 43% of 110 patients with femoral shaft fractures treated with intramedullary nailing had > 10° of torsion, with 19% exceeding 15° [16]. Since the prevalence of relevant malrotation (≥ 15°) in femoral nonunion -as presented in this study- is in line with these numbers after acute femoral fracture fixation one might hypothesize that malrotation per se does not necessarily contribute to nonunion development. Nevertheless, the high prevalence of clinically relevant malrotation in nonunion underlines the importance of a careful clinical and CT-based imaging assessment of torsional deformity to correct this as part of the nonunion treatment.

Also, the results suggest that there is no tendency towards either internal or external torsional deformities. This is consistent with previous literature: Mansouri-Tehrani et al. reported in a cohort of 140 patients with isolated femoral shaft fracture treated with antegrade intramedullary nailing, 56% external and 44% internal torsion deformities [26]. Jaarsma et al. found in a cohort of 76 patients with unilateral femoral shaft fractures treated with intramedullary nailing that of the patients who had deformity exceeding ≥ 15°, 16% were external and 12% were internal malrotated [25]. Moreover, Jaarsma et al. noted that patients with an external rotational deformity tend to report more complaints than those with an internal rotational deformity [25]. From a clinical perspective, these findings underline the importance of obtaining bilateral CT-scans in patients with femoral shaft nonunion. This is the gold standard as the anatomy of the contralateral femur is a better reference for natural anatomical deformity restoration than the population average and therefore helps optimize surgical planning [27]. In this study, bilateral CT-scans of the complete femur were used. An alternative method would be to use only slices restricted to the femoral neck and distal condyles, as this is sufficient to measure torsion and length discrepancy while reducing radiation exposure and making preoperative screening more practical, although this does not allow assessment of coronal and sagittal plane deformity.

Also, relying on plain radiographs or clinical judgment alone may underestimate the prevalence and extent of rotational malalignment, thereby missing opportunities for deformity correction [28, 29]. This is also relevant as intraoperative assessment of femoral rotation is challenging. Techniques such as using the true lateral view, the neck horizontal angle, and the lesser trochanter profile are widely used to guide reduction in acute femoral fracture cases, yet these are prone to inaccuracy and may fail to prevent/correct malrotation [28, 29]. Accordingly, awareness of deformity at time of nonunion treatment (and how to correct this) is important to reduce the risk of a symptomatic nonunion subsequently becoming a symptomatic malunion.

In addition to torsional deformities, the results showed that 16% of patients had a leg length discrepancy of ≥ 20 mm, a threshold generally considered clinically relevant due to the symptoms that may occur such as lower back pain and osteoarthritis [23]. Although the mean difference between the affected and contralateral femur was modest (6 mm), vigilance to this discrepancy seems relevant for substantial outliers.

For the identification of coronal and sagittal plane deformities, thresholds of ≥ 5° and ≥ 10° have been established, based on the distribution (mean, standard deviation, range) reported coronal and sagittal plane variation throughout literature. These values indicated that 10/32 (31%) patients had a ≥ 5° difference in coronal plane angulation, and 5/32 (16%) had a ≥ 10° difference in sagittal plane angulation. On average, apex anterior angulation was slightly reduced in the affected femora compared with the contralateral side and did reach statistical significance. Similarly, coronal alignment showed a trend toward greater variability in the affected limb, with a wider range of varus/valgus malalignment, in which varus (25%) was far more common than valgus (6%); a known risk factor for failure of osteosynthesis and also nonunion development [30, 31]. The wider ranges observed in nonunion femora indicate that individualized assessment remains important. Notably, failure to correct significant angular deformities may still have long-term consequences, including an unstable mechanical environment which may lead to persisting nonunion.

Interestingly, torsional deformities were observed not only in AO type C (comminuted), but also in type A (simple) and B (wedge) fractures. These results show that also simpler fracture patterns can still lead to torsional deformities, and it underlines that every type of fracture needs preoperative attention as intraoperative control may be insufficient.

There are several limitations in this study. First, despite covering a nearly 10-year inclusion period, the study sample remains relatively small, which reduces statistical power and may obscure subtle associations between fracture type, fixation method, and deformity. However, it addresses a clear gap in the literature by specifically evaluating torsional deformities in femoral diaphyseal nonunions, while also considering length discrepancy and observed coronal and sagittal malalignments. Future research should aim to assess whether deformity relates to patient-reported outcomes and the development of nonunion. Implant choice and long-term consequences such as osteoarthritis could further refine clinical decision-making and optimize surgical planning. Second, although interobserver reliability in this study was excellent for torsional and length measurements, coronal plane deformity assessment showed only moderate agreement. This suggests that measurements in the coronal plane remain more challenging. This may be explained by less distinct landmarks in the diaphyseal region and/or hardware artifacts obscuring the cortical margins along which the measurements were taken. Nevertheless, bilateral CT-based measurements allowed for precise and reproducible assessment across multiple planes and were performed by three observers independently which gave the most accurate results possible. Third, in 26 patients the CT-scan was of insufficient quality (no contralateral or no full femur CT) which may have influenced the results, as these patients may have had less severe deformity and could therefore result in skewed prevalences in this cohort.

Taken together, these findings underline the usefulness of preoperative bilateral CT imaging in assessment of deformity in femoral shaft nonunions and reinforce the critical role of accurate rotational and length control during primary fracture fixation.

## Conclusion

About 1 in 3 patients who undergo diaphyseal femoral nonunion repair have a clinically relevant torsional deformity. Additionally, a femoral length discrepancy of ≥ 20 mm was observed in approximately 1 out of 6 patients. A small, but significant, decrease in apex anterior angulation was found, and most outliers in the coronal plane were in varus. The study underlines the importance of being vigilant about recognizing deformity in femoral nonunions.

## Supplementary Information

Below is the link to the electronic supplementary material.


Supplementary Material 1


## Data Availability

The data used in this study consist of confidential patient information and cannot be made publicly available due to privacy, ethical, and legal restrictions.
