# Supplementary material for: What is the rate and degree of deformity in femoral diaphyseal nonunion? A CT-based retrospective cohort study
Source: Eur J Trauma Emerg Surg. 2026 Aug 28;52(1):272. doi: 10.1007/s00068-026-03314-1 (PMC13525014; doi:10.1007/s00068-026-03314-1)
Supplement: Supplementary file 1 [file 68_2026_3314_MOESM1_ESM.pdf]

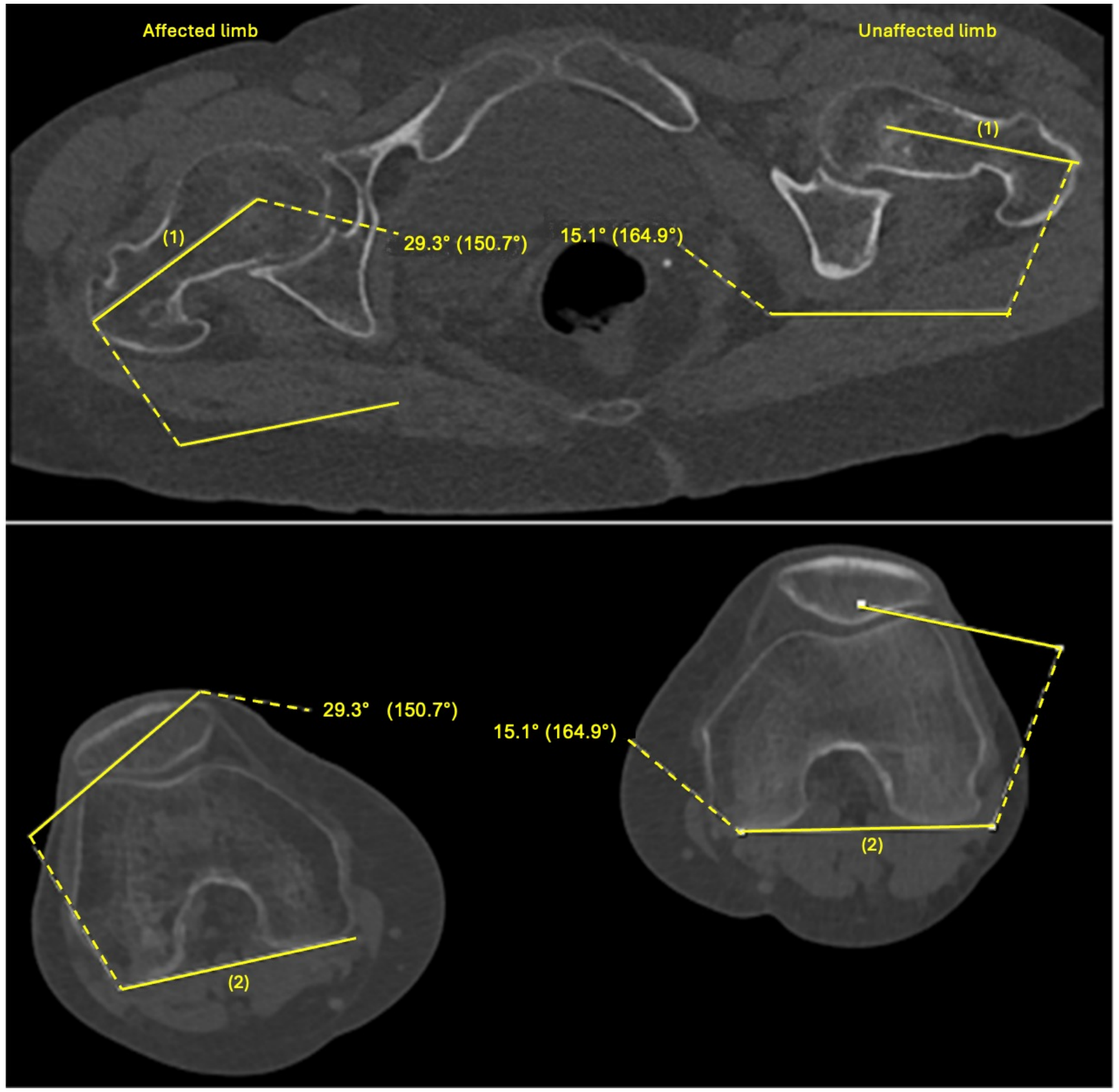

Online resource 1 Axial plane measurements

Affected limb: 29.3°, unaffected limb:15.1°: 14.2° difference in torsion (internal)

(1): Line drawn across the femoral neck

(2): Line drawn across the posterior distal femoral condyles

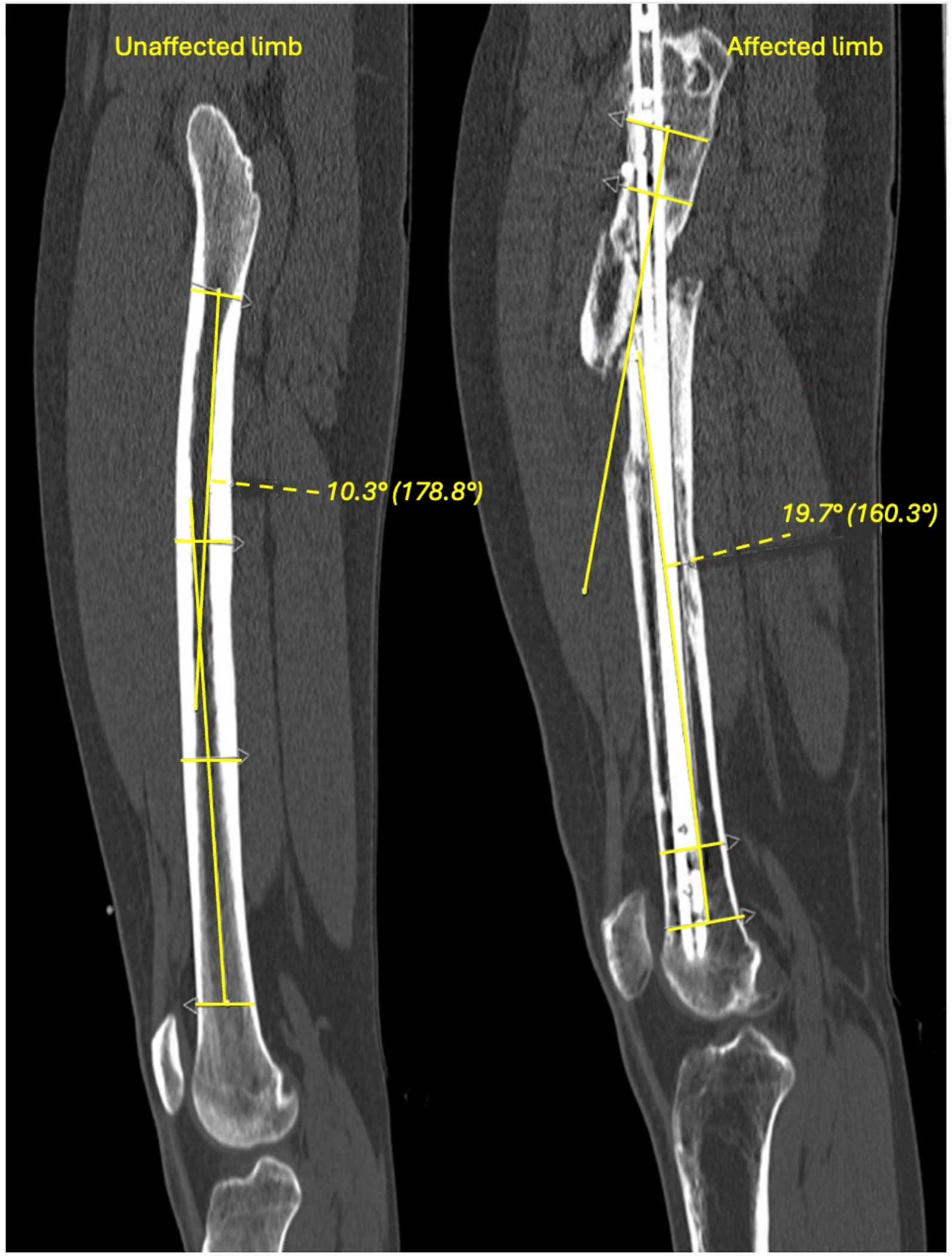

Online resource 2 Sagittal plane measurements

Affected limb:19.7°, unaffected limb:10.3°: 9.4° difference in apex anterior angulation

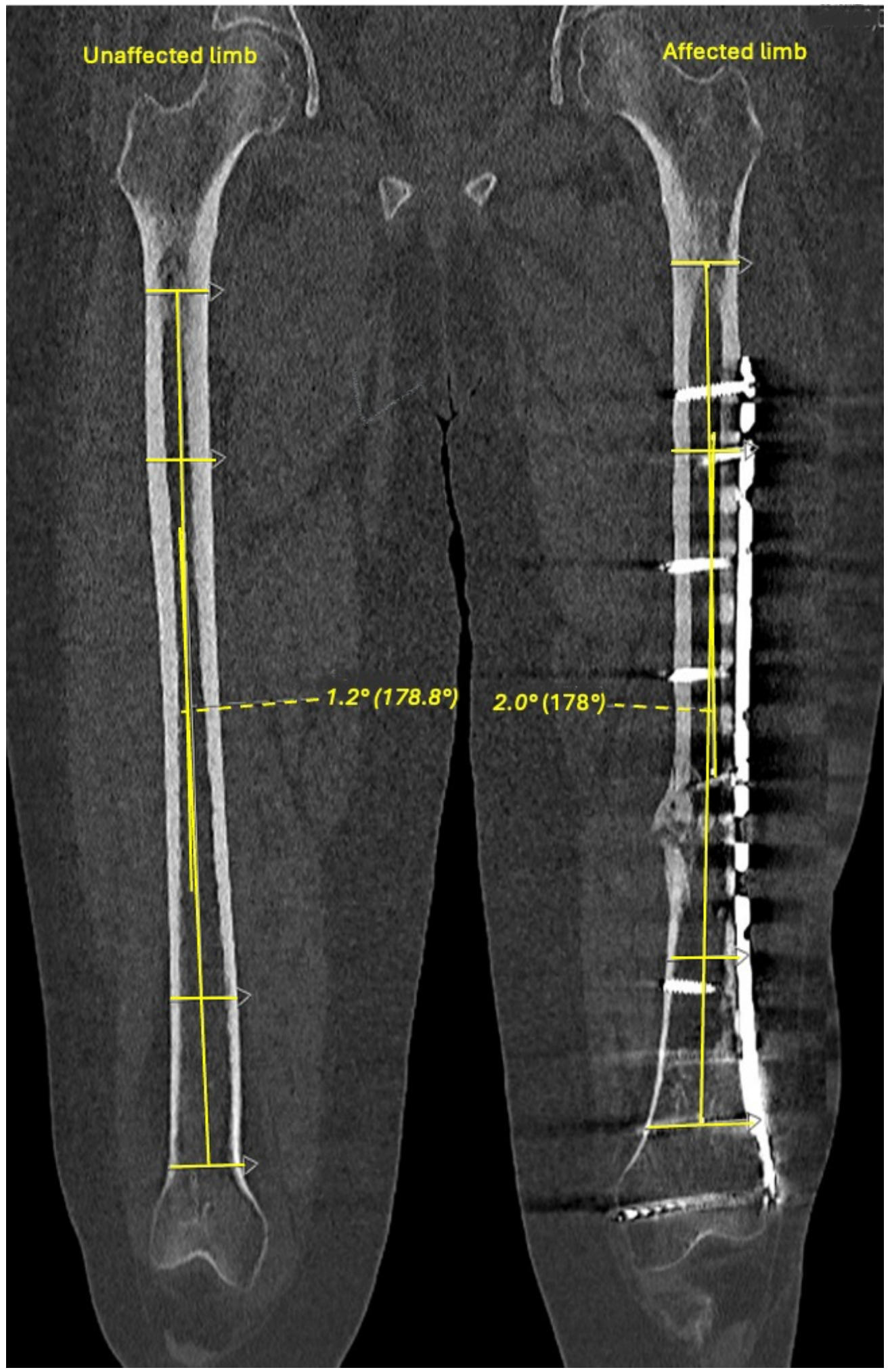

Online resource 3 Coronal plane measurements

Affected limb: 2.0° varus, unaffected limb: 1.2° varus: 0,8° difference in varus

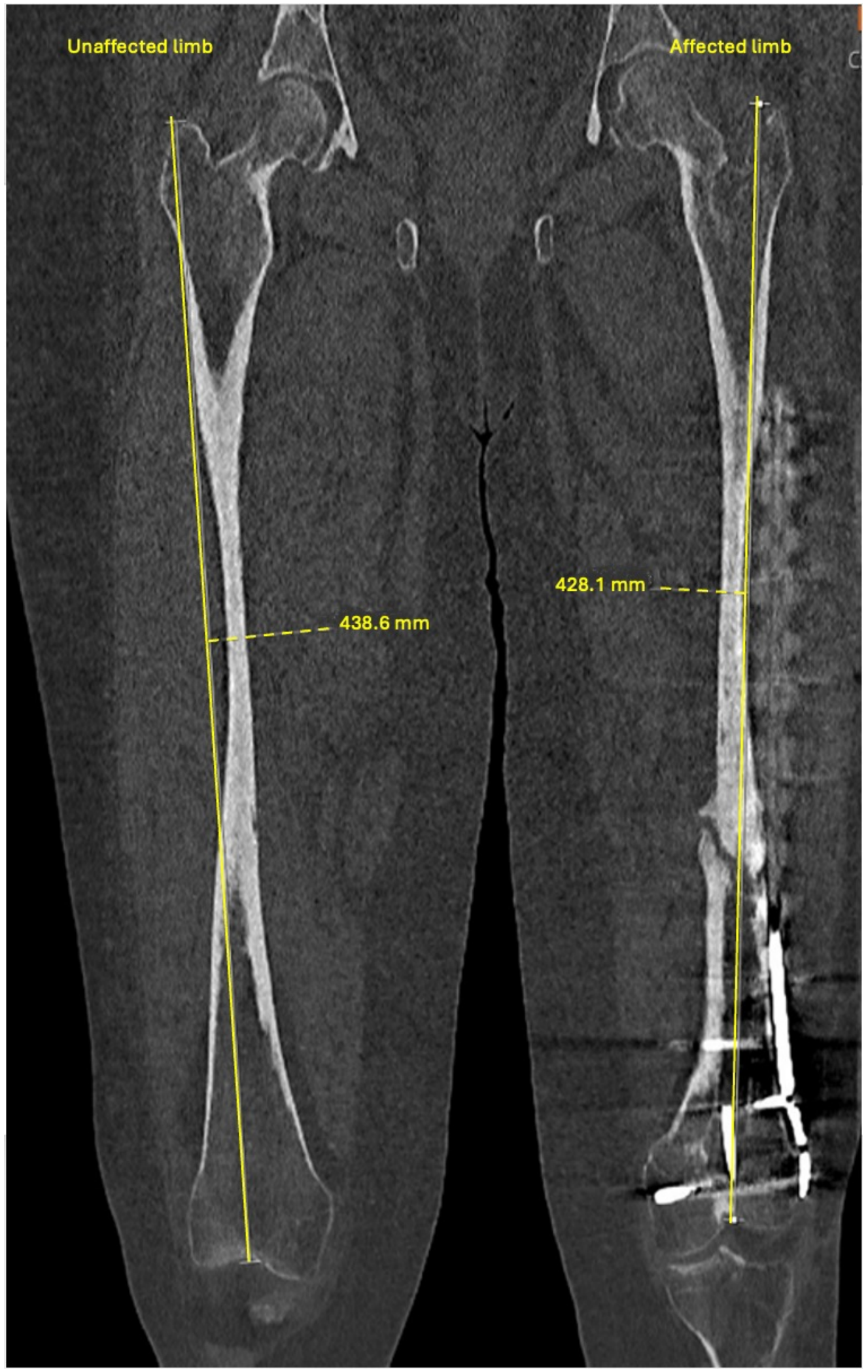

Online resource 4 Femoral length measurements

Affected limb: 428.1 mm, unaffected limb: 438.6: *shortening of 10.5 mm of affected leg*

Measurement Plane	Limb	ICC (95% CI)	p-value	Interpretation
**Axial**	Affected	0.96 (0.93–0.98)	< 0.001	Excellent
	Unaffected	0.94 (0.87–0.97)	< 0.001	Excellent
**Sagittal**	Affected	0.87 (0.77–0.93)	< 0.001	Good
	Unaffected	0.79 (0.63–0.89)	< 0.001	Good
**Coronal**	Affected	0.75 (0.75–0.87)	< 0.001	Good
	Unaffected	0.67 (0.42–0.82)	< 0.001	Moderate
**Length**	Affected	0.99 (0.98–0.99)	< 0.001	Excellent
	Unaffected	0.99 (0.98–0.99)	< 0.001	Excellent

Online resource 5 Two-way random effects model with absolute agreement, based on the guidelines by Koo & Li, 2016.[1]

1 Koo, T. K. & Li, M. Y. A Guideline of Selecting and Reporting Intraclass Correlation Coefficients for Reliability Research. *J Chiropr Med* **15**, 155-163 (2016). https://doi.org/10.1016/j.jcm.2016.02.012
